# Knockdown of lncRNA ZNRD1‐AS1 inhibits progression of bladder cancer by regulating miR‐194 and ZEB1

**DOI:** 10.1002/cam4.3373

**Published:** 2020-08-30

**Authors:** Zhixiang Gao, Shidong Li, Xufeng Zhou, Huali Li, Shasha He

**Affiliations:** ^1^ Department of Imaging And Magnetic Response the Luoyang Central Hospital Affiliated to Zhengzhou University Luoyang China

**Keywords:** bladder cancer, EMT, miR‐194, proliferation, ZEB1, ZNRD1‐AS1

## Abstract

**Background:**

Bladder cancer (BC) is a common urinary neoplasm with high incidence worldwide. Long noncoding RNA zinc ribbon domain containing 1 antisense RNA 1 (ZNRD1‐AS1) has been reported to be upregulated in BC. However, the exact role of ZNRD1‐AS1 as well as its mechanism remains poorly understood.

**Methods:**

Zinc ribbon domain containing 1 antisense RNA 1, and its potential downstream genes microRNA‐194 (miR‐194) and zinc finger E‐box binding homeobox 1 (ZEB1) levels were detected via quantitative real‐time polymerase chain reaction or western blot. Cell proliferation, migration, invasion, and epithelial‐mesenchymal transition (EMT) were detected to assess the influences of ZNRD1‐AS1, miR‐194 and ZEB1 on BC cells by colony formation, cell counting kit‐8 (CCK‐8), transwell analysis or western blot. The relationship between miR‐194 and ZNRD1‐AS1 or ZEB1 was analyzed by luciferase activity analysis. The xenograft experiment was performed to assess the function of ZNRD1‐AS1 in vivo.

**Results:**

Zinc ribbon domain containing 1 antisense RNA 1level was upregulated in BC. ZNRD1‐AS1 silence repressed proliferation, migration, invasion and EMT in BC cells. MiR‐194 was identified as a target of ZNRD1‐AS1, and miR‐194 upregulation repressed proliferation, migration, invasion, and EMT by ZNRD1‐AS1 sponging. ZEB1 was targeted via miR‐194 and its interference impeded proliferation, migration, invasion, and EMT. Moreover, ZNRD1‐AS1 regulated ZEB1 expression via miR‐194. Besides, inhibition of ZNRD1‐AS1 attenuated tumor growth by miR‐194/ZEB1 axis in vivo.

**Conclusion:**

Knockdown of ZNRD1‐AS1 suppressed BC cell development in vitro and in vivo via targeting miR‐194 to regulate ZEB1, indicating a novel avenue for treatment of BC.

## Graphical abstract

ZNRD1‐AS1 expression was elevated and miR‐194 was reduced in BC tissues and cells. Abrogation of ZNRD1‐AS1 suppressed proliferation, migration, invasion, and EMT by sponging miR‐194. ZEB1 interference impeded progression of BC cells and regulated by ZNRD1‐AS1 and miR‐194. Besides, inhibition of ZNRD1‐AS1 attenuated the tumor growth by regulating miR‐194 and ZEB1 in vivo. Collectively, depletion of ZNRD1‐AS1 inhibited BC progression in vitro and in vivo by acting as a ceRNA for miR‐194 to regulate ZEB1, providing a novel avenue for treatment of BC.

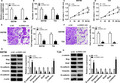

## 1 INTRODUCTION

Bladder cancer (BC) is a common malignancy with more than 430, 000 cases each year all over the world.^1^ Tobacco smoking is the main risk factor for BC with high incidence and mortality.^2^ In recent years, prognosis and prediction of BC have gained more attentions, while the survival remains poor.^3^ Hence, it is urgent to explore novel therapeutic targets as well as the molecular mechanism.

Emerging evidence suggests that mRNA‐, microRNA (miRNA)‐, and long noncoding RNA (lncRNA)‐based biomarkers play important roles in diagnosis, prognosis, and development of BC.^4^ Moreover, lncRNAs have been reported as therapeutic targets to be implicated in proliferation, invasiveness, apoptosis, and cell cycle in BC.^5^ For example, lncRNA membrane‐associated guanylate kinase inverted‐2 antisense RNA 3 (MAGI2‐AS3) suppresses proliferation, migration, and invasion via modulating miR‐15b‐5p and coiled‐coil domain containing 19 (CCDC19) in BC.^6^ Knockdown of lncRNA plasmacytoma variant translocation 1 (PVT1) inhibits growth, migration, and invasion via regulating miR‐31 and cyclin‐dependent kinase 1 (CDK1) in BC.^7^ LncRNA zinc ribbon domain containing 1 antisense RNA 1 (ZNRD1‐AS1) has been suggested to participate in the development of cancers, including cervical cancer and hepatocellular carcinoma.^8^, ^9^ Furthermore, previous report indicates ZNRD1‐AS1 is upregulated in BC tissues in comparison to normal samples.^10^ Nevertheless, the exact role of ZNRD1‐AS1 in BC progression as well as its mechanism remain elusive.

miRNA, a group of small noncoding RNA, plays essential roles in diagnosis, prognosis, monitoring and therapy of BC patients.^11^ Moreover, miRNA has an important impact on proliferation, apoptosis, migration, invasion, and epithelial‐mesenchymal transition (EMT) in BC.^12^ Increasing efforts have indicated miR‐194 as tumor inhibitor to repress cell proliferation, migration, and invasion and induce apoptosis in pancreatic cancer and prostate cancer.^13^, ^14^ Furthermore, microarray analysis suggests that miR‐194 is ectopic in BC.^15^ Nevertheless, the role and mechanism of miR‐194 in BC development are largely unclear.

Zinc finger E‐box binding (ZEB) family is a main factor of EMT, which exhibits a pivotal role in EMT.^16^ ZEB1, a member of ZEB family, has key roles in EMT, migration and invasion in human cancers.^17^ In addition, ZEB1 is reported to be upregulated in BC.^18^, ^19^ Bioinformatics assay provides the putative binding sequence of miR‐194 and ZNRD1‐AS1 or ZEB1. But there is no direct evidence in support of this prediction.

In this study, we hypothesized ZNRD1‐AS1 might regulate BC cell development via functioning as a competing endogenous RNA (ceRNA) for miR‐194 to mediate ZEB1 in BC cells. First, we explored the effect of ZNRD1‐AS1 on proliferation, migration, invasion and EMT, and then the relationships between ZNRD1‐AS1 and miR‐194, miR‐194, and ZNRD1‐AS1 as well as the interaction among them in BC cells were investigated. Also, the role of ZNRD1‐AS1 in BC in vivo was further explored.

## 2 MATERIALS AND METHODS

### 2.1 Clinical samples

Thirty BC patients were enrolled in this study from the Luoyang Central Hospital Affiliated to Zhengzhou University. The tumor and adjacent normal control (NC) tissues from subjects were collected and stored at −80°C. All participants without the history of other therapy before the surgery have provided the written informed consent. This research was approved via the Research Ethics Committee of Luoyang Central Hospital Affiliated to Zhengzhou University.

### 2.2 Cell culture and transfection

The human bladder epithelium cell line (SVHUC‐1) and BC cell lines (RT4, UMUC3, T24, SW780, 5637, and RT112) were provided via American Tissue Culture Collection (Manassas, VA, USA) and grew in DMEM medium (Gibco, Carlsbad, CA, USA) plus 10% fetal bovine serum (FBS; Gibco), and 1% penicillin/streptomycin (Invitrogen, Carlsbad, CA, USA) at 37°C and 5% CO_2_.

Small interfering RNA (siRNA) against ZNRD1‐AS1 (si‐ZNRD1‐AS1), siRNA against ZEB1 (si‐ZEB1), siRNA negative control (si‐NC), pcDNA, ZNRD1‐AS1 overexpression vector (pcDNA‐ZNRD1‐AS1), miR‐194 mimic (miR‐194), miRNA negative control (miR‐NC), miR‐194 inhibitor (in‐miR‐194), and miRNA inhibitor negative control (in‐miR‐NC) were generated via Genepharma (Shanghai, China). Cell transfection was conducted in SW780 and T24 cells for 48h by Lipofectamine 2000 (Invitrogen) following the manufacturer's instructions.

### 2.3 Quantitative real‐time polymerase chain reaction (qRT‐PCR)

RNA was isolated with TRIzol (Invitrogen) and reversely transcribed via TaqMan microRNA Reverse Transcription Kit (Applied Biosystems, Foster City, CA, USA) or M‐MLV Reverse Transcription Kit (Thermo Fisher, Wilmington, DE, USA). qRT‐PCR was carried out with SYBR green (Applied Biosystems). The relative expression of RNA was analyzed with U6 small RNA or β‐actin as reference via 2^−ΔΔCt^ method.^20^ The primers included: miR‐194 (Forward, 5’‐ATGGACCTGGGGCCAG CGAAG‐3’; Reverse, 5’‐TCTGGCCTGGGAGCGTCG‐3’), U6 (Forward, 5’‐GCTTC GGCAGCACATATACTAAAAT‐3’; Reverse, 5’‐CGCTTCACGAATTTGCGTGTCA T‐3’), ZNRD1‐AS1 (Forward, 5’‐TCCTAGGATTGCTGCAGGTC‐3’; Reverse, 5’‐C TATTGCCTGGATCCCATGT‐3’), ZEB1 (Forward, 5’‐AGCGAG GTAAAGTTGC GTCT‐3’; Reverse, 5’‐AGGTTTTCTGGGCCATACCG‐3’), β‐actin (Forward, 5’‐CA GCCTTCCTTCTTGGGTAT‐3’; Reverse, 5’‐TGGCATAGAGGTCTTTACGG‐3’).

### 2.4 Colony formation

After the transfection, SW780 and T24 cells (600 cells/well) were added into six‐well plates for 14d. The medium was refreshed every 3d. Colonies were fixed via methanol (Sigma, St. Louis, MO, USA) for 30min and next dyed with 0.01% crystal violet (Sigma) for 15min, followed via observation with a microscope (Olympus, Tokyo, Japan).

### 2.5 Cell proliferation

Cell proliferation was analyzed via cell counting kit 8 (CCK‐8) (Yeasen, Shanghai, China). In brief, SW780 and T24 cells (1, 000 cells/well) were placed into 96‐well plates at 37°C for 0, 24, 48, 72 or 96 hours. Then cells were incubated with 10μL of CCK‐8 solution at 37°C for 2 hours. The absorbance was examined at 450 nm with a microplate reader (Bio‐Rad, Hercules, CA, USA).

### 2.6 Transwell analysis

Cell migrated and invasive abilities were assessed via transwell assay. In brief, SW780 and T24 cells (4 × 10^4^ cells/well) in 100 μL of non‐serum DMEM medium were seeded in the upper chambers (Corning, Corning, NY, USA), and 600 μL of medium with 10% FBS was filled to the lower chambers. Following the culture for 24 hours, migrated cells were fixed with 4% paraformaldehyde (Sigma) and dyed with 0.1% crystal violet, followed via observation using a microscope with five randomly selected fields. For cell invasion, the transwell chambers were pre‐treated with Matrigel (BD, San Jose, CA, USA) and experiment was conducted according to the same approach.

### 2.7 Western blot

Proteins were extracted via RIPA solution (Beyotime, Shanghai, China) and the concentration was analyzed via BCA kit (Beyotime) following centrifugation. After the denaturation, 30 μg samples were separated on SDS‐PAGE and transferred to polyvinylidene difluoride membranes (Sigma). After blocking in 1% bovine serum albumin (Sigma) for 1h, the membranes were interacted with primary antibodies overnight at 4°C and secondary antibody for 2h. The antibodies for β‐catenin (ab32572, 1:5000 dilution), E‐cadherin (ab15148, 1:500 dilution), Slug (ab106077, 1:1000 dilution), Snail (ab216347, 1:1000 dilution), N‐cadherin (ab76057, 1:1000 dilution), Vimentin (ab16700, 1:1000 dilution), ZEB1 (ab203829, 1:500 dilution) or β‐actin (ab8227, 1:3000 dilution) and horseradish peroxidase (HRP)‐conjugated secondary antibody (ab6721, 1:10000 dilution) were purchased from Abcam (Cambridge, UK). β‐actin served as a reference and the blots were visualized via enhanced chemiluminescence (Beyotime).

### 2.8 Luciferase activity analysis

The potential complementary sequence of miR‐194 and ZNRD1‐AS1 or ZEB1 was searched via DIANA tools or TargetScan. The sequence of ZNRD1‐AS1 or ZEB1 3’‐UTR containing wild‐type (WT) or mutant‐type (MUT) binding sequence was inserted into the pGL3 vectors (Promega, Madison, WI, USA) to synthesize luciferase reporter vectors (ZNRD1‐AS1‐WT, ZNRD1‐AS1‐MUT, ZEB1‐WT, or ZEB1‐MUT). SW780 and T24 cells were co‐transfected with 20ng luciferase reporter vector, 15ng control vector and 40nM miR‐194, miR‐NC, in‐miR‐194, or in‐miR‐NC via Lipofectamine 2000 for 48 hours. The luciferase activity was measured using luciferase assay system (Promega).

### 2.9 Xenograft model

BALB/c nude mice (male, 5‐week‐old) were provided via Vital River (Beijing, China) and maintained in a pathogen‐free condition with a 12 hours light/dark cycle and free access to water and food for 1 week. The experiment was performed with the approval of the Animal Research Committee of "XXXXX." SW780 cells (2 × 10^6^) stably transfected with the lentiviral vectors with si‐ZNRD1‐AS1 or si‐NC (GeneCopoeia, Rockville, MD, USA) were subcutaneously injected into the nude mice (n = 7/group). Tumor volume was detected every 7 days, and calculated using the formula: width^2^ × length ×0.5. After 5 weeks, the animals were sacrificed and tumor samples were weighed and then used for the further analyses.

### 2.10 Statistical analysis

Data of all analyses were exhibited as the mean ± standard deviation (SD) from three independent experiments. The correlation between the abundance of miR‐194, ZNRD1‐AS1, ZEB1 in BC tissues was tested via Spearman rank correlation. The statistical differences between groups were tested via student's *t* test or one‐way analysis of variance via GraphPad Prism 5 (GraphPad Inc, La Jolla, CA, USA). *P* < .05 was considered as statistically significant.

## 3 RESULTS

### 3.1 ZNRD1‐AS1 expression was enhanced in BC

**Figure 1:**
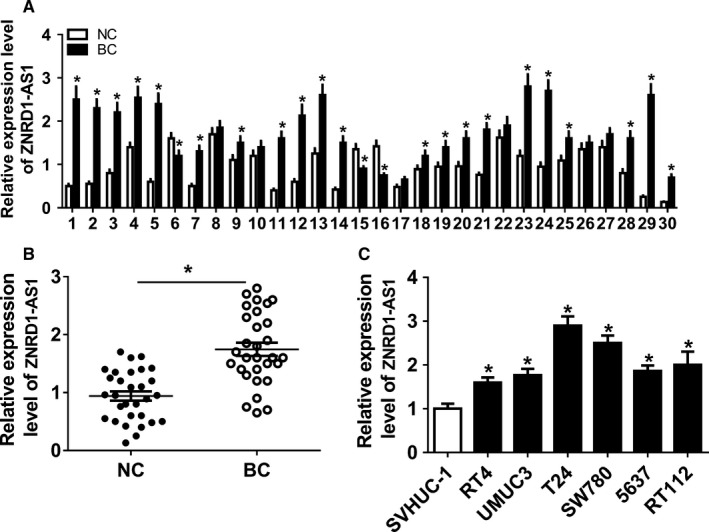
ZNRD1‐AS1 expression was enhanced in BC. (A) The levels of ZNRD1‐AS1 was measured in each subject by qRT‐PCR. (B) The expression of ZNRD1‐AS1 was detected in BC and NC tissues by qRT‐PCR. (C) The abundance of ZNRD1‐AS1 was examined in BC cells by qRT‐PCR. *P < .05

To analyze the potential role of ZBRD1‐AS1 in BC, its abundance was first examined in BC tissue samples and cell lines. Results showed that ZNRD1‐AS1 abundance displayed increased trend in BC tissue samples in comparison to NC group except for reduction of three cases and little alteration of six cases (Figure 1A). Taken as a whole, ZNRD1‐AS1 abundance was evidently increased in BC tissue samples compared with BC group (Figure 1B). Similarly, BC cells also showed higher abundance of ZNRD1‐AS1 than normal SVHUC‐1 cells (Figure 1C). Thus, SW780 and T24 cells with relatively higher ZNRD1 levels were used for further studies.

### 3.2 Knockdown of ZNRD1‐AS1 suppressed proliferation, migration, invasion, and EMT in BC cells

**Figure 2:**
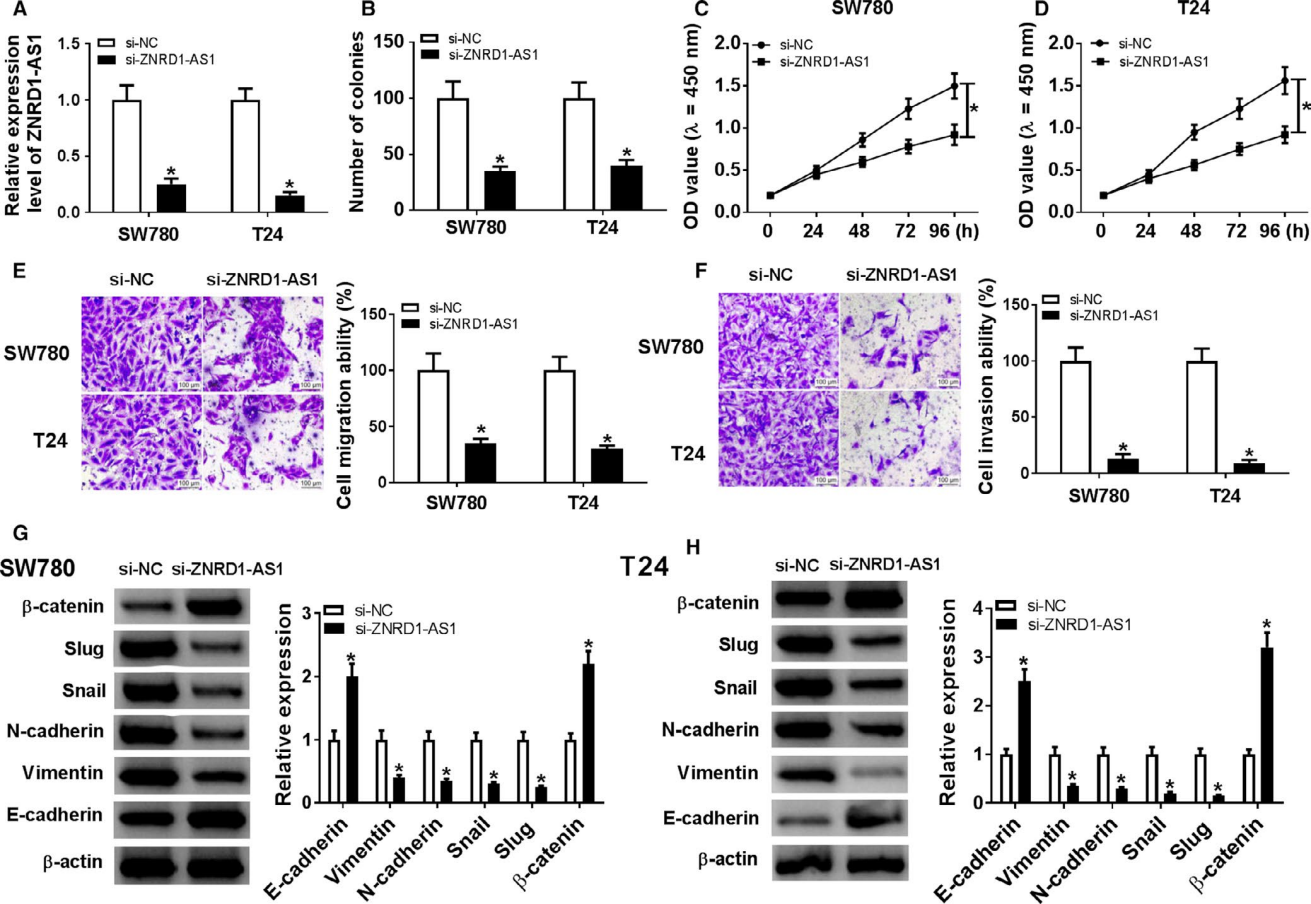
Knockdown of ZNRD1‐AS1 inhibited proliferation, migration, invasion, and EMT in BC cells. (A) The expression of ZNRD1‐AS1 was measured in SW780 and T24 cells transfected with si‐ZNRD1‐AS1 or si‐NC by qRT‐PCR. The number of colonies (B), proliferation (C and D), migration (E), invasion (F), and EMT (G and H) were detected in SW780 and T24 cells transfected with si‐ZNRD1‐AS1 or si‐NC by colony formation, CCK‐8, transwell or western blot assays, respectively. *P < .05

To analyze the function of ZNRD1‐AS1 on BC cell development, SW780 and T24 cells were transfected with si‐ZNRD1‐AS1 or si‐NC. ZNRD1‐AS1 abundance was effectively declined in SW780 and T24 cells via transfection of si‐ZNRD1‐AS1 (Figure 2A). ZNRD1‐AS1 knockdown remarkably declined the number of colonies in SW780 and T24 cells (Figure 2B). Moreover, inhibition of ZNRD1‐AS1 significantly suppressed cell proliferation at 48, 72, and 96h in SW780 and T24 cells (Figure 2C, D). Furthermore, abrogation of ZNRD1‐AS1 notably impeded cell migration and invasion (Figure 2E, F). Besides, obvious increase of β‐catenin and E‐cadherin and loss of Vimentin, N‐cadherin, Snail, and Slug protein levels were displayed in SW780 and T24 cells with silence of ZNRD1‐AS1 (Figure 2G, H).

### 3.3 miR‐194 was bound to ZNRD1‐AS1

**Figure 3:**
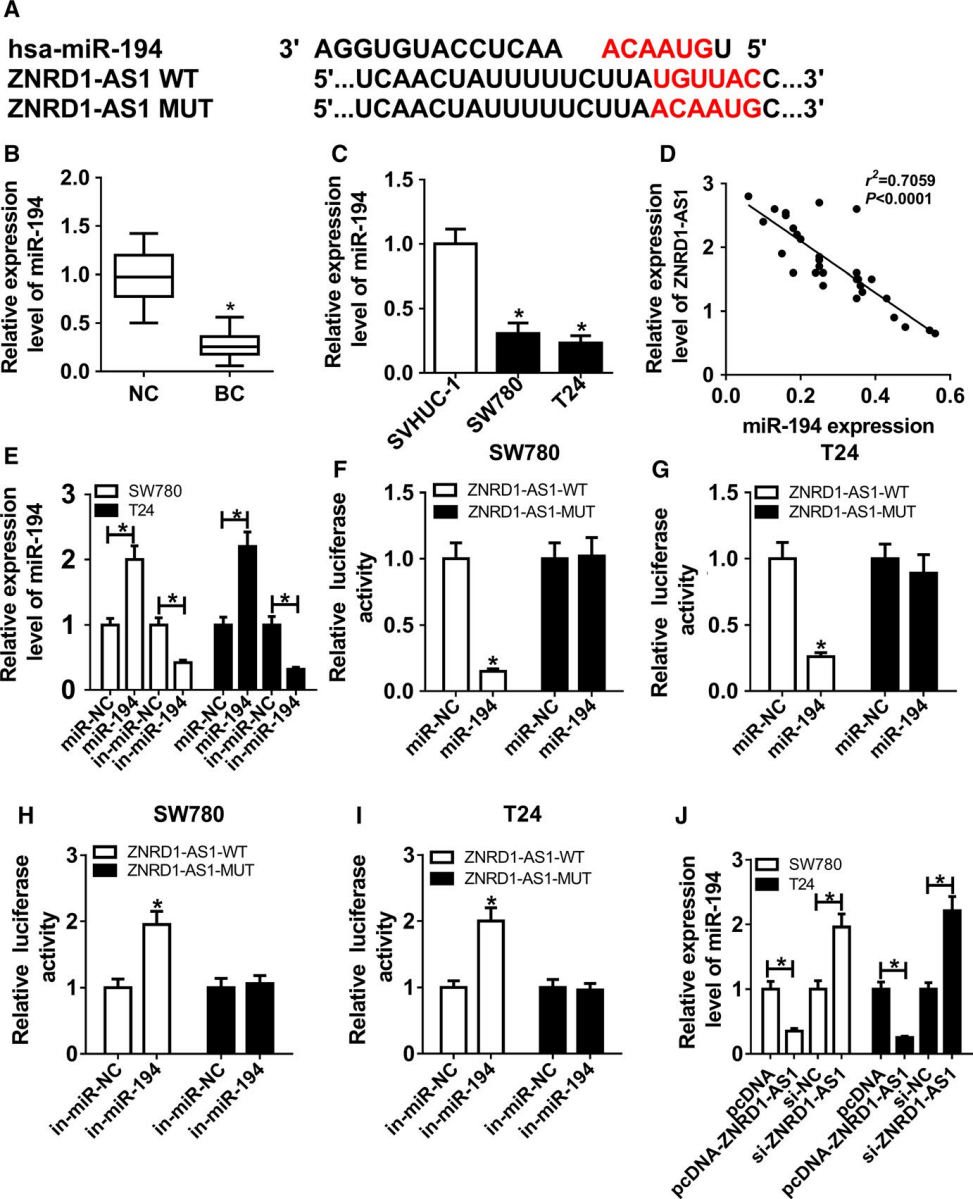
miR‐194 was bound to ZNRD1‐AS1. (A) The putative binding sites of ZNRD1‐AS1 and miR‐194 were predicted by DIANA tools. (B and C) The expression of miR‐194 was measured in BC tissues and cells by qRT‐PCR. (D) The correlation between the expressions of ZNRD1‐AS1 and miR‐194 in BC tissues was analyzed by Spearman rank correlation. (E) The abundance of miR‐194 was detected in SW780 and T24 cells transfected with miR‐194, miR‐NC, in‐miR‐194, or in‐miR‐NC by qRT‐PCR. (F‐I) Luciferase activities were analyzed in SW780 and T24 cells co‐transfected with ZNRD1‐AS1‐WT or ZNRD1‐AS1‐MUT and miR‐194, miR‐NC, in‐miR‐194 or in‐miR‐NC. (J) The expression of miR‐194 was measured in SW780 and T24 cells transfected with pcDNA, pcDNA‐ZNRD1‐AS1, si‐NC, or si‐ZNRD1‐AS1 by qRT‐PCR. *P < .05

Seeing that lncRNA participates in ceRNA regulatory network, the potential bound miRNAs of ZNRD1‐AS1 were explored. Bioinformatics analysis provided the putative binding sequence of ZNRD1‐AS1 and miR‐194 and we conducted the WT or MUT luciferase reporter vector (Figure 3A). miR‐194 expression was detected in BC tissues and cell lines. Results showed that miR‐194 level was abnormally declined in BC samples and cells in comparison to their corresponding control, respectively (Figure 3B, C). Moreover, the expression of ZNRD1‐AS1 was negatively related with miR‐194 abundance in BC tissues (*r* ^2^ = 0.7059, *P* < .0001) (Figure 3D). Subsequently, SW780 and T24 cells were transfected with miR‐194, miR‐NC, in‐miR‐194 or in‐miR‐NC. miR‐194 abundance was effectively enhanced via transfection of miR‐194 and inhibited via transfection of in‐miR‐194 (Figure 3E). In addition, miR‐194 overexpression induced obvious reduction of luciferase activity in SW780 and T24 cells with transfection of ZNRD1‐WT, but its efficacy was abated in response to ZNRD1‐AS1 group (Figure 3F, G). However, deficiency of miR‐194 exhibited an opposite effect on luciferase activity (Figure 3H, I). Besides, miR‐194 expression was significantly impaired in SW780 and T24 cells transfected with pcDNA‐ZNRD1‐AS1 and elevated in cells with transfection of si‐ZNRD1‐AS1 (Figure 3J).

### 3.4 ZNRD1‐AS1 sponged miR‐194 to regulate progression of BC cells

**Figure 4:**
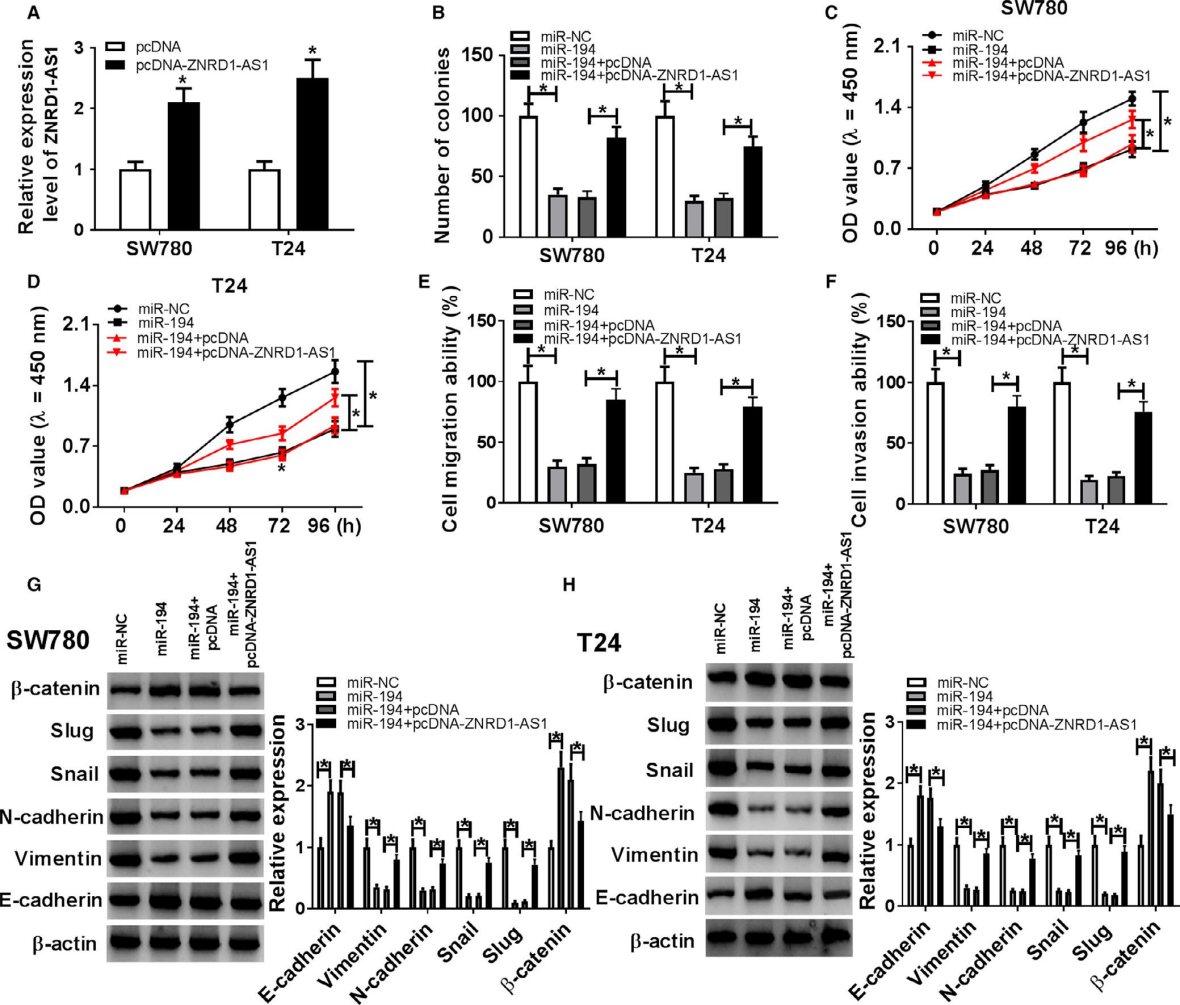
ZNRD1‐AS1 regulated proliferation, migration, invasion, and EMT by sponging miR‐194 in BC cells. (A) The expression of ZNRD1‐AS1 was measured in SW780 and T24 cells transfected with pcDNA or pcDNA‐ZNRD1‐AS1 by qRT‐PCR. The number of colonies (B), proliferation (C and D), migration (E), invasion (F), and EMT (G and H) were detected in SW780 and T24 cells transfected with miR‐NC, miR‐194, miR‐194 + pcDNA, or miR‐194 + pcDNA‐ZNRD1‐AS1 by colony formation, CCK‐8, transwell, or western blot assays, respectively. *P < .05

To explore whether ZNRD1‐AS1 affected the progression of BC cells by sponging miR‐194, SW780, and T24 cells were transfected with miR‐NC, miR‐194, miR‐194, and pcDNA or pcDNA‐ZNRD1‐AS1. As displayed in Figure 4A, ZNRD1‐AS1 expression was markedly increased in cells with transfection of pcDNA‐ZNRD1‐AS1 compared with that treated with pcDNA vector alone. The number of colonies was remarkably suppressed via miR‐194 overexpression, which was reversed via addition of ZNRD1‐AS1 (Figure 4B). Moreover, cell proliferation was notably limited by upregulation of miR‐194 and attenuated by introduction of ZNRD1‐AS1 in SW780 and T24 cells (Figure 4C, D). Additionally, accumulation of miR‐194 evidently reduced migration and invasion of SW780 and T24 cells, which was weakened by addition of ZNRD1‐AS1 (Figure 4E, F). Besides, overexpression of miR‐194 resulted in great increase of E‐cadherin and β‐catenin and inhibition of Vimentin, N‐cadherin, Snail and Slug in SW780 and T24 cells, which was alleviated via enrichment of ZNRD1‐AS1 (Figure 4G, H).

### 3.5 ZEB1 was targeted via miR‐194

**Figure 5:**
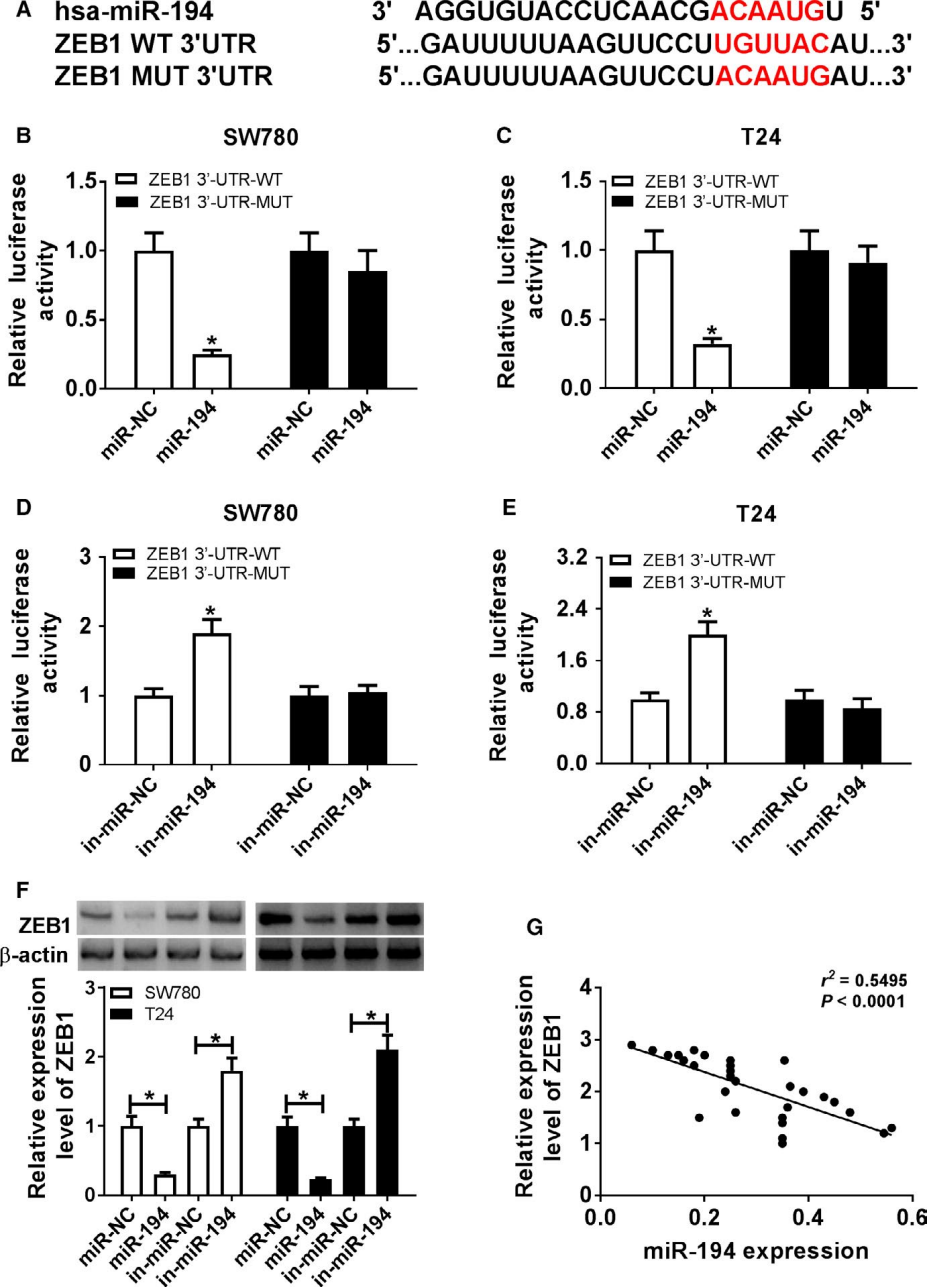
ZEB1 was a target of miR‐194. (A) The potential binding sites of miR‐194 and ZEB1 were probed by TargetScan. (B‐E) Luciferase activities were measured in SW780 and T24 cells co‐transfected with ZEB1‐WT or ZEB1‐MUT and miR‐194, miR‐NC, in‐miR‐194, or in‐miR‐NC. (F) The expression of ZEB1 protein was detected in SW780 and T24 cells transfected with miR‐194, miR‐NC, in‐miR‐194, or in‐miR‐NC by western blot. (G) The relationship between miR‐194 and ZEB1 mRNA levels in BC tissues was analyzed by Spearman rank correlation. *P < .05

To test how miR‐194 participated in BC cell progression, the targets of miR‐194 were probed via TargetScan. Bioinformatics analysis showed the putative binding sequence of miR‐194 and ZEB1 (Figure 5A). Luciferase activity analysis was conducted to validate the prediction. miR‐194 overexpression resulted in obvious reduction of luciferase activity in SW780 and T24 cells with transfection of ZEB1‐WT, while it could not influence the activity in ZEB1‐MUT group (Figure 5B, C). Nevertheless, miR‐194 knockdown had an opposite impact on the luciferase activity (Figure 5D, E). Then the influence of miR‐194 on ZEB1 protein level was measured via western blot. Results showed that miR‐194 overexpression greatly declined ZEB1 protein level, but its knockdown promoted ZEB1 expression (Figure 5F). Furthermore, the abundance of ZEB1 mRNA was negatively associated with miR‐194 abundance in BC tissues (*r* ^2^ = 0.5495, *P* < .0001) (Figure 5G).

### 3.6 Interference of ZEB1 suppressed progression of BC cells

**Figure 6:**
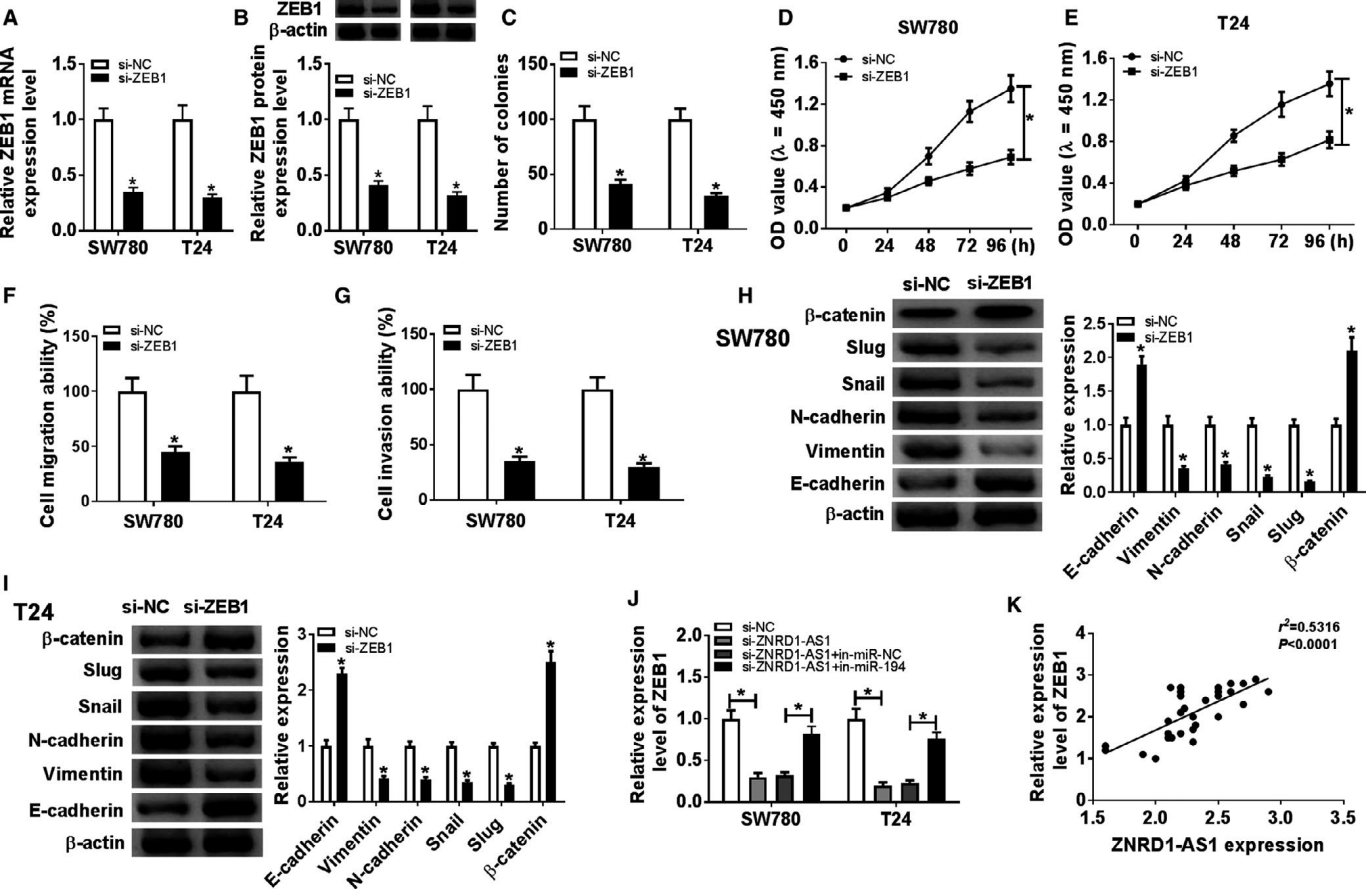
Inhibition of ZEB1 suppressed proliferation, migration, invasion, and EMT in BC cells. (A and B) The expression of ZEB1 was measured at mRNA and protein levels in SW780 and T24 cells transfected with si‐ZEB1 or si‐NC by qRT‐PCR or western blot. The number of colonies (C), proliferation (D and E), migration (F), invasion (G), and EMT (H and I) were measured in SW780 and T24 cells transfected with si‐ZEB1 or si‐NC by colony formation, CCK‐8, transwell or western blot assays, respectively. (J) The abundance of ZEB1 protein was detected in SW780 and T24 cells transfected with si‐NC, si‐ZNRD1‐AS1, si‐ZNRD1‐AS1 + in‐miR‐NC, or si‐ZNRD1‐AS1 + in‐miR‐194 by qRT‐PCR. (K) The associated with between ZEB1 and ZNRD1‐AS1 expressions in BC tissues was analyzed by Spearman rank correlation. *P < .05

To assess the function of ZEB1 in BC cell progression, SW780 and T24 cells were transfected with si‐ZEB1 or si‐NC. After the transfection, ZEB1 expression was effectively decreased at mRNA and protein levels in SW780 and T24 cells via transfection of si‐ZEB1 (Figure 6A, B). Furthermore, inhibition of ZEB1 significantly impeded the colony formation and proliferation in SW780 and T24 cells (Figure 6C‐E). In addition, downregulation of ZEB1 strikingly protected against cell migration and invasion (Figure 6F, G). Compared with control group, ZEB1 knockdown significantly enhanced the expressions of E‐cadherin and β‐catenin but declined the abundances of Vimentin, N‐cadherin, Snail, and Slug (Figure 6H, I). Besides, ZEB1 expression was examined in cells with transfection of si‐NC, si‐ZNRD1‐AS1, si‐ZNRD1‐AS1, and in‐miR‐NC or in‐miR‐194. Results revealed that the mRNA level of ZEB1 was aberrantly inhibited by ZNRD1‐AS1 knockdown and rescued by miR‐194 inhibition in SW780 and T24 cells (Figure 6J). And ZEB1 mRNA expression was positively associated with ZNRD1‐AS1 expression (*r* ^2^ = 0.5316, *P* < .0001) (Figure 6K).

### 3.7 Inhibition of ZNRD1‐AS1 attenuated xenograft tumor growth via modulating miR‐194 and ZEB1 in vivo

**Figure 7:**
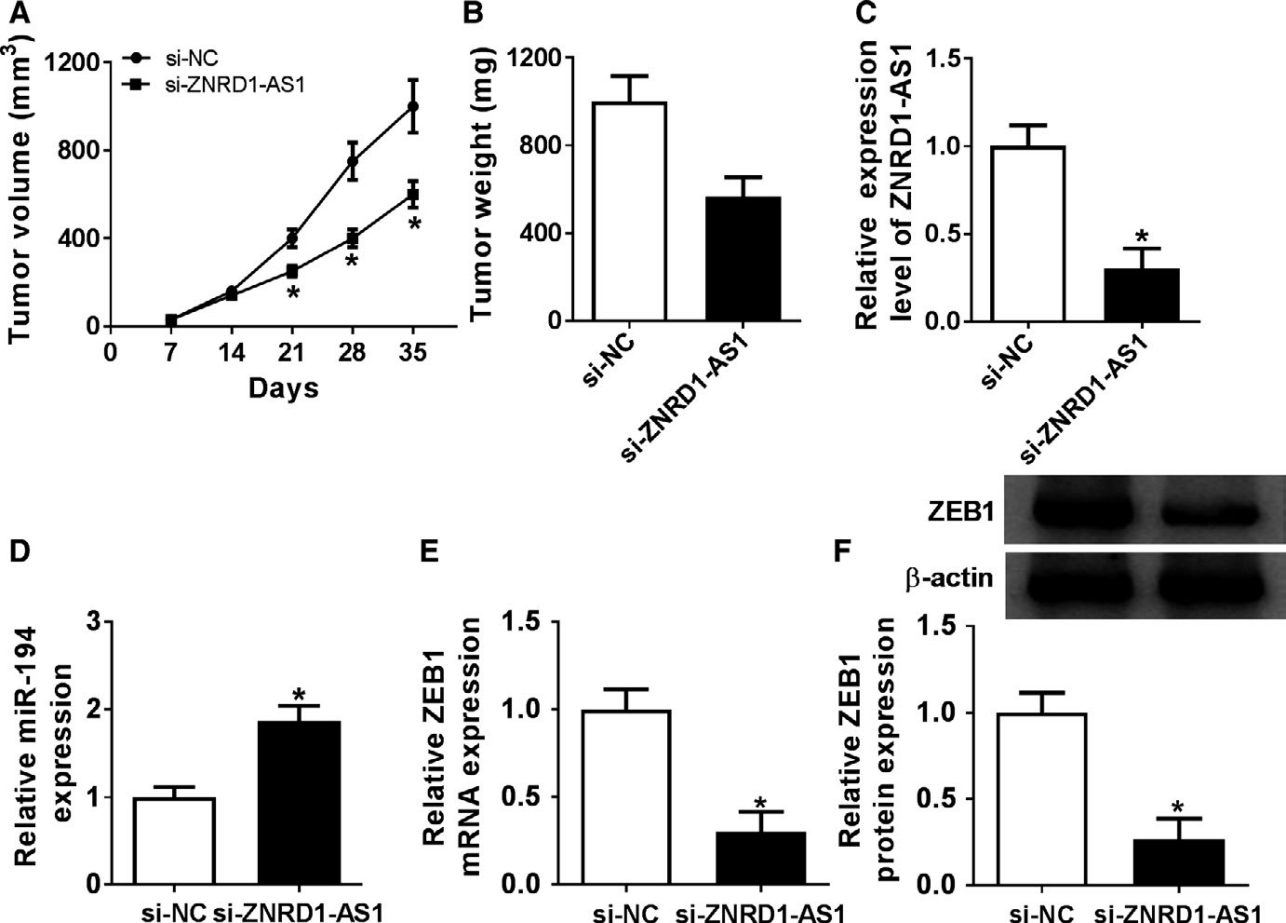
Interference of ZNRD1‐AS1 attenuated xenograft tumor growth by regulating miR‐194 and ZEB1. (A) Tumor volume was measured every week in si‐NC or si‐ZNRD1‐AS1 group. (B) Tumor weight was detected at ending point. The abundances of ZNRD1‐AS1 (C), miR‐194 (D), ZEB1 mRNA (E), and protein (F) were measured in tumor tissues by qRT‐PCR or western blot, respectively. *P < .05

To further evaluate function of ZNRD1‐AS1 in BC in vivo, SW780 cells with stable transfection of si‐ZNRD1‐AS1 or si‐NC were introduced into nude mice. Results showed that knockdown of ZNRD1‐AS1 significantly inhibited tumor volume as well as weight compared with si‐NC group (Figure 7A, B). Then ZNRD1‐AS1, miR‐194 and ZEB1 abundances were examined in tumor tissues. The abundance of ZNRD1‐AS1 was obviously declined in si‐ZNRD1‐AS1 group compared with si‐NC group (Figure 7C). Moreover, the level of miR‐194 was prominently elevated in si‐ZNRD1‐AS1 group (Figure 7D). Besides, si‐ZNRD1‐AS1 group exhibited lower abundance of ZEB1 than si‐NC group (Figure 7E, F).

## 4 DISCUSSION

Previous study has reported that abnormally expressed lncRNAs may function as important biomarkers for the treatment of BC.^21^ In this study, we found that ZNRD1‐AS1 expression was elevated in BC tissues and cells, which is also consistent with previous report.^10^ These data implied that ZNRD1‐AS1 might have important roles in BC development. Here we first provided the view that ZNRD1‐AS1 knockdown inhibited progression of BC through acting as a ceRNA for miR‐194 to mediate ZEB1.

Accruing literatures have revealed that inhibition of epithelial markers (E‐cadherin and β‐catenin) as well as elevation of mesenchymal markers (Vimentin, N‐cadherin, Snail, and Slug) contributed to EMT, which was associated with development of cancers.^22^, ^23^ In the present study, knockdown of ZNRD1‐AS1 led to increase of E‐cadherin and β‐catenin and reduction of Vimentin, N‐cadherin, Snail, and Slug, which indicated that ZNRD1‐AS1 abrogation blocked EMT in BC cells. Moreover, depletion of ZNRD1‐AS1 suppressed proliferation, migration, and invasion in BC cells. These results uncovered that ZNRD1‐AS1 might act as an oncogene in BC. However, the mechanism of ZNRD1‐AS1 participating in BC progression remains unknown. Former works have elucidated that lncRNAs may serve as ceRNAs to regulate the development of cancers.^24^, ^25^ Furthermore, certain lncRNAs have been identified to play a vital role in BC progression via regulating ceRNA network.^26^ Hence, the downstream target of ZNRD1‐AS1 need be further explored.

Online software and luciferase reporter assay verified miR‐194 as a target of ZNRD1‐AS1. Moreover, ZNRD1‐AS1 negatively regulated miR‐194 expression. MiR‐194 has been reported to function as tumor suppressor to inhibit proliferation, migration, invasion, drug resistance, and EMT in varying cancers, such as laryngeal squamous cell carcinoma, glioma, and prostate cancer.^27^, ^28^, ^29^ Moreover, miR‐194 was found to be downregulated in BC, and the inhibitory effect of miR‐194 on BC cells was also demonstrated.^30^ Similarly, we found that miR‐194 abundance was reduced in BC, and miR‐194 overexpression impeded proliferation, migration, invasion, and EMT in BC cells. These data also indicated miR‐194 as a tumor suppressor in BC. In addition, the anti‐tumor effect was reversed by addition of ZNRD1‐AS1, which revealed ZNRD1‐AS1 regulated BC progression by sponging miR‐194.

Functional miRNAs were known to regulate targets abundances in many conditions. Sever such reports have disclosed many targets of miR‐194 in different cell lines. For instance, miR‐194 inhibited cell proliferation, migration, and invasion via decreasing transforming growth factor alpha in colorectal cancer.^31^ Moreover, miR‐194 suppressed osteosarcoma cell proliferation and migration but contributed to apoptosis via regulating cadherin 2.^32^ In our study, TargetScan predicted ZEB1 as a downstream target of miR‐194. A previous study has revealed that miR‐194 reduced paclitaxel resistance of ovarian cancer cells via targeting ZEB1.^33^ However, whether this regulatory network could affect BC cell development remains unknown. Here we also confirmed the correlation of miR‐194 and ZEB1 in BC cells. Besides, ZNRD1‐AS1 regulated ZEB1 expression via miR‐149.

Previous study suggested that ZEB1 was enhanced in BC and contributed to cell proliferation, migration and EMT by functioning as a target of lncRNA regulator of reprogramming (ROR) in BC cells.^34^ Furthermore, ZEB1 signaling has been reported to be associated with metastasis and EMT in BC.^35^ Additionally, ZEB1 was indicated as a target of miR‐23b and its silence suppressed proliferation, migration, and invasion in BC.^36^ Consistent with these studies, we also showed that depletion of ZEB1 suppressed cell proliferation, migration, invasion, and EMT in BC cells. Finally, in vivo experiment showed that knockdown of ZNRD1‐AS1 alleviated tumor growth by upregulating miR‐194 and downregulating ZEB1 in xenograft model. However, there is inadequacy in our study. Whether ZNRD1‐AS1/miR‐194/ZEB1 axis regulates BC cell progression via some signaling pathways is still unclear. Thus, the potential signaling pathway participating in BC progression is needed to be explored in further study.

## 5 CONCLUSION

Zinc ribbon domain containing 1 antisense RNA 1 abundance was elevated and miR‐194 was reduced in BC tissues and cells. Abrogation of ZNRD1‐AS1 suppressed proliferation, migration, invasion, and EMT via sponging miR‐194. ZEB1 interference impeded progression of BC cells and regulated by ZNRD1‐AS1 and miR‐194. Besides, inhibition of ZNRD1‐AS1 attenuated tumor growth via modulating miR‐194 and ZEB1 in vivo. Collectively, depletion of ZNRD1‐AS1 inhibited BC progression in vitro and in vivo by acting as a ceRNA for miR‐194 to regulate ZEB1, indicating a new avenue for treatment of BC.

## ETHICS STATEMENT

All the patients involved in this study signed informed consents. This study was supported by the ethics committee of the Luoyang Central Hospital Affiliated to Zhengzhou University.

## CONFLICTS OF INTEREST

None declare.

## Data Availability

All the data used and analyzed during this study was available from the corresponding author on the request.
